# Novel Perspectives in Immune Checkpoint Inhibitors and the Management of Non-Alcoholic Steatohepatitis-Related Hepatocellular Carcinoma

**DOI:** 10.3390/cancers14061526

**Published:** 2022-03-16

**Authors:** Landon L. Chan, Stephen L. Chan

**Affiliations:** 1 Department of Oncology, Princess Margaret Hospital, Hong Kong, China; landonchan@ha.org.hk; 2 Department of Clinical Oncology, Sir YK Pao Centre for Cancer, Prince of Wales Hospital, The Chinese University of Hong Kong, Hong Kong, China; 3 State Key Laboratory of Translational Oncology, The Chinese University of Hong Kong, Hong Kong, China

**Keywords:** hepatocellular carcinoma, NASH-HCC, immune checkpoint inhibitors

## Abstract

**Simple Summary:**

Hepatocellular carcinoma (HCC) is a huge worldwide healthcare burden. The incidence of non-viral causes of hepatocellular carcinoma, such as non-alcoholic steatohepatitis (NASH), is rising. The introduction of immune checkpoint inhibitors (ICI) has led to a paradigm shift in the systemic treatment of HCC. However, not all patients can benefit from ICI. Recent studies have suggested that the response to ICI may allude to the underlying aetiology of HCC, such as NASH. Our review aims to summarise the latest evidence of ICI in advanced HCC, elaborates on the controversy surrounding the use of ICI in NASH-HCC, and discusses potential biomarkers that can be used to predict responses to ICI in advanced HCC.

**Abstract:**

Immune checkpoint inhibitors have revolutionised the systemic treatment of advanced hepatocellular carcinoma. Although phase III trials, testing single agent nivolumab and pembrolizumab, failed to meet their primary endpoints, the combination of atezolizumab and bevacizumab has demonstrated a remarkable objective response and unprecedented survival benefits, replacing sorafenib as the standard first-line treatment for advanced hepatocellular carcinoma. Despite these successes observed in immune checkpoint inhibitors in the management of advanced hepatocellular carcinoma, not all patients responded to treatment, which has led to the search of risk factors and biomarkers that could predict the response to immune checkpoint inhibitors. Recent translational studies have begun to shed light on the impact of an underlying liver disease, namely NASH, which might affect the response to immune checkpoint inhibitors. In addition, antidrug-antibody and gene expression assays have demonstrated promises in predicting the response to immune checkpoint inhibitors. In this article, we will provide an overview of the use of ICI in the management of advanced HCC, review the evidence that surrounds the recent controversy regarding NASH-HCC, and discuss potential biomarkers that predict the response to immune checkpoint inhibitors.

## 1. Introduction

Hepatocellular carcinoma (HCC) is a global healthcare challenge with increasing incidence and mortality [1,2]. HCC is the sixth-most-common cancer and the third leading cause of cancer mortality globally in 2020, resulting in more than 900,000 new cases and 830,000 deaths [1]. The major risk factors for HCC include chronic viral infection (e.g., hepatitis B, hepatitis C), metabolic syndromes (e.g., type 2 diabetes, obesity, non-alcoholic fatty liver disease (NAFLD), NASH) and its related lifestyle (e.g., smoking). While the incidence of viral HCC has declined due to the widespread vaccination program and increased public awareness, the incidence of NAFLD/NASH-HCC has risen steadily, possibly due to the changing prevalence of obesity and diabetes [2].

Although early HCC can be managed with curative surgery, ablation or liver transplantation, the majority of HCC patients present with unresectable or recurrent disease requiring systemic treatment. It is estimated that approximately 50–60% of HCC patients will eventually be treated with systemic therapy [2]. Conventionally, HCC is not responsive to chemotherapy and has a dismal prognosis. In 2008, the introduction of sorafenib, a multi-kinase inhibitor, became the first breakthrough in systemic treatment for HCC. Both the SHARP trial [3] and Asia-Pacific trial [4] demonstrated that there was a small but significant median overall survival (OS) benefit with sorafenib in advanced HCC (SHARP: sorafenib: 10.7 months vs. the placebo: 7.9 months, HR: 0.69; Asia-Pacific: sorafenib: 6.5 months vs. the placebo: 4.2 months, HR: 0.68). Unfortunately, further drug development in advanced HCC was met with failure for a decade, with four major trials testing other multi-kinase inhibitors demonstrating negative results [5,6,7,8]. In 2013, a phase III randomised trial (*n* = 1074) evaluating sunitinib against sorafenib resulted in early termination due to futility and safety reasons. The median OS was significantly shorter with sunitinib compared with sorafenib (7.9 vs. 10.2 months, HR: 1.30) [5]. In the same year, another phase III randomised trial (*n* = 1155) evaluating brivanib against sorafenib also failed to reach its primary endpoint of non-inferiority OS. The median OS was 9.9 months for sorafenib and 9.5 months for brivanib (HR: 1.06) [6]. Two years later, in 2015, linifanib was compared with sorafenib in the treatment of advanced HCC in a phase III trial (*n* = 1035). This trial was also negative because it failed to meet its primary endpoint of non-inferiority OS (linifanib: 9.1 months vs. sorafenib: 9.8 months, HR: 1.05) [7]. In the same year, another phase III randomised trial (*n* = 720) comparing sorafenib plus erlotinib with sorafenib alone in advanced HCC also reported negative result. The trial reported that the addition of erlotinib did not improve the median OS (sorafenib plus erlotinib: 9.5 months vs. sorafenib: 8.5 months, HR: 0.93) [8]. In 2018, lenvatinib became the second drug that demonstrated effectiveness in treating advanced HCC in the first-line setting [9]. In the non-inferiority REFLECT trial, patients treated with lenvatinib had similar median OS (lenvatinib: 13.6 months vs. sorafenib: 12.3 months, HR: 0.92) but attained a higher median progression-free survival (PFS) (lenvatinib: 7.4 months vs. sorafenib: 3.7 months) and objective response rate (ORR) (lenvatinib: 24.1% vs. sorafenib: 9.2%). After failing sorafenib, three multi-kinase inhibitors (e.g., regorafenib, cabozantinib and ramucirumab) demonstrated OS benefits in the second-line setting [10,11,12]. However, the ORRs of these agents were low, at ~10%, and tolerance was poor in general (Table 1).

The recent introduction of immune checkpoint inhibitors (ICI) has revolutionised the management in oncology across multiple cancer types, including HCC [13,14,15,16]. Evidence of ICI in the management of advanced HCC emerged in 2017. In a phase I/II dose escalation and expansion CheckMate-040 trial, single-agent nivolumab (an antibody against PD-1) brought about an unprecedentedly high ORR at 20% in advanced HCC, with a safe and manageable side-effect profile, regardless of the underlying aetiology [17]. In the year that follows, pembrolizumab (an antibody against PD-1), also demonstrated a similar ORR, at 17%, in the phase II KEYNOTE-224 trial [18]. Despite the initially promising results of phase I/II trials of nivolumab and pembrolizumab, both agents did not meet their primary endpoints at statistical significance in their respective phase III trials (CheckMate-459 and KEYNOTE-240), demonstrating a median OS of 16.4 months and 13.9 months, respectively [19,20]. This has led to the search for combination strategies in enhancing the response to ICI, including combination with another ICI [21,22], an anti-VEGF/multi-kinase inhibitor [23,24,25] or locoregional therapy [26,27,28]. Another line of active research is to investigate risk factors and biomarkers that could predict response to ICI [29,30].

Recently, a paper indicated that the use of anti-PD-1 treatment in the context of NASH may potentially accelerate hepatocarcinogenesis [31]. This finding was further substantiated by a meta-analysis performed by the same group, demonstrating the response to ICI that was apparently restricted to viral HCC [31]. The meta-analysis (*n* = 1656) included three large randomised trials of patients (CheckMate-459, IMbrave150 and KEYNOTE-240) with advanced HCC treated with ICI. In the overall population, ICI improved survival (HR: 0.77, 95%; CI: 0.63–0.94). Survival was also improved in the viral HCC subgroup (HR: 0.64, 95%; CI: 0.48–0.84) but not in the non-viral HCC subgroup (HR: 0.92, 95%; CI: 0.77–1.11).

With this background, it is timely to review the current evidence of ICI in advanced HCC and the controversy that surrounds it in the management of NASH-HCC.

## 2. Immune Surveillance, Immune Microenvironment, and the Immune Checkpoints

### 2.1. Immunoediting

The concept of immune surveillance can be traced back to more than a centennial ago. In 1909, Paul Ehrlich formulated the hypothesis that the human body constantly generated neoplastic cells that could be eradicated by the immune system [32]. However, this was not proven due to inadequate knowledge and experimental tools. In the 1960s and 1970s, Lewis Thomas and Sir Frank Macfarlane Burnet independently proposed what has presently become the foundation of the theory of immune surveillance. They stated that tumour-associated antigens can be recognised and targeted by the immune system to prevent carcinogenesis, similarly to graft rejections [33]. This concept was supported by experiments in the mouse model, demonstrating that genetically identical mice could be immunised against transplants of syngeneic tumour cells [34]. In 2002, Dunn et al. proposed a refinement of the concept of immune surveillance, called ‘immunoediting’, which could be classified into three phases: elimination, equilibrium, and escape [35]. In the elimination phase, cancer cells with tumour-associated antigens are recognised by the immune system and are eliminated. The equilibrium phase is the intermediate step, in which the immune system iteratively selects and promotes the generation of tumour cell variants with increasing capacities to evade immune surveillance. In this phase, tumour cell growth is still under control. In the final escape phase, it is characterised by uncontrolled tumour growth, due to the survival advantage sculpted by the immune system in an immunocompetent host [35].

### 2.2. The Immune Microenvironment in the Liver

It is important to understand that the pathogenesis of HCC, regardless of the underlying aetiology, stems from chronic inflammation. It is essential to recognise that the immune microenvironment in the liver is distinctive to other organs, in order to prioritise biomarkers for drug development and treatment strategies. The liver has a unique blood supply, with the hepatic arterial blood joining the portal venous blood and draining into the hepatic veins, within a structure called the liver sinusoid. A large spectrum of microbes, microbe-associated molecular patterns (MAMPs) and damage-associated molecular patterns (DAMPs) continuously shower the liver sinusoids from the gut through the portal blood [36]. While these foreign molecules are constantly recognised and removed by tissue residential immune cells, such as the Kupffer cells (KCs), immunotolerance is maintained through the intricate interactions between the basal pro-inflammatory molecules (e.g., IL-2, IL-7, IL-12, IL-15 and IFN-γ) produced by the hepatic stellate cells (HSCs), NK cells, NKT-cells and γδ T-cells, and counter-balanced by anti-inflammatory cytokines (e.g., IL-10, IL-13, transforming growth factor beta (TGF-β)) produced by the myeloid-derived suppressor cells (MDSCs), regulatory T-cells (Tregs), liver sinusoidal endothelial cells (LSEC) and KCs [37,38].

Under conditions of chronic inflammation, this balance is lost and tips towards the pro-inflammatory state, resulting in increased hepatocytes turnover, compensatory proliferation, acquisition of mutations and malignant changes [38]. Eventually, HCC develops under the background of exhausted and dysfunctional effector cytotoxic T-cells. This is accompanied by the immunosuppressive tumour microenvironment, which is bathed with tumour-associated macrophages (TAMs), Tregs and MDSCs.

**Figure 1:**
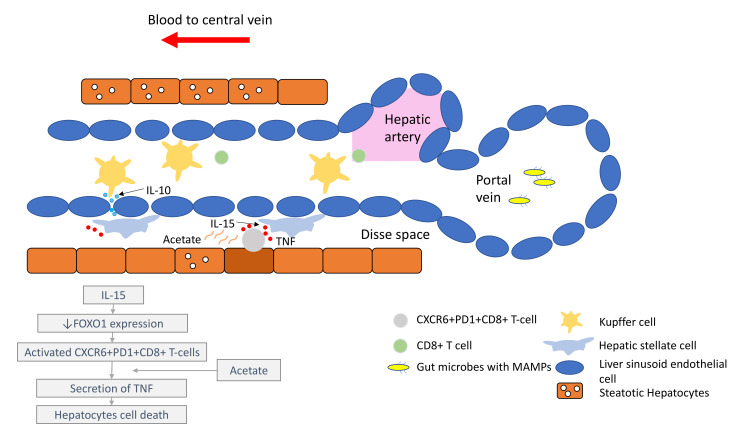
Liver immune microenvironment in NASH patients. In NASH patients, liver immune tolerance is lost and tips towards pro-inflammation. The IL-15 triggers decreased FOXO1 expression and activates the residential CD8+PD1+ T-cells. These residential T-cells, upon encounter with the acetate secreted by steatotic hepatocytes, release TNF, which triggers hepatocyte cell death through a FAS–ligand-dependent manner. This process is thought to bring about the NASH to NASH-HCC transition.

Comprehensive multiomics and single-cell analyses of human HCC tissues provided support of the presence of immunosuppressive cell populations in the tumour microenvironment, which fostered the growth of HCC [39,40]. Amongst them, TAMs are the most well studied. TAMs consist of KCs and blood/bone borne monocyte-derived macrophages. TAMs promote HCC progression in several ways, including the secretion of IL-10 and other immunosuppressive cytokines, promotion of angiogenesis, recruitment of Tregs and IL-17-expressing CD4+ T helper 17 cells, expression of inhibitory immune-checkpoint ligand PD-L1 and induction of proliferation signalling pathways [16,38,41]. Together with a high number of MDSCs residing in the liver, which secrete immunosuppressive molecules, such as vascular endothelial growth factor (VEGF), TGF-β and arginase that suppress T-cells activation [38], a strong immunosuppressive milieu is created, leading to immune escape and enabling uncontrolled tumour growth (Figure 1).

### 2.3. The Immune Checkpoints, CTLA-4 and PD-1

Amongst the most studied immune checkpoints to date are CTLA-4 and PD-1 [42,43]. These immune checkpoints have been successfully targeted by drugs to restore immunosurveillance for tumour cell killing. CTLA-4 is a negative regulator of T-cell activity. It acts in the priming phase in the draining lymph nodes, where T-cell activation takes place [44]. T-cell activation requires two signals: the first signal occurs when the naïve T-cells encounter their cognate antigen presented by the antigen-presenting cells on their MHC molecule. This activates T-cells but is inadequate to maintain their activation unless a second signal is provided. The second signal is provided by the CD80/86 molecules on the antigen-presenting cells and the CD28 molecules on naïve T-cells. This second signal lowers the threshold of T-cell activation significantly and enables T-cells to differentiate and engage in clearing foreign antigens, such as bacteria or tumour cells. Once the T-cells become activated, they begin to express an inhibitory molecule to tamper down the immune response to avoid overactivity. This is achieved by CTLA-4’s higher affinity for CD80/86 than CD28, impairing effective T-cell activation. Furthermore, CTLA-4 is constitutively expressed on Tregs and thus plays an essential role in maintaining the immunosuppressive environment within the tumour microenvironment [45]. Consequently, the ICI targeting CTLA-4 aims to reinvigorate T-cell activation to counteract the ability of tumour cells to escape immunosurveillance.

**Figure 2:**
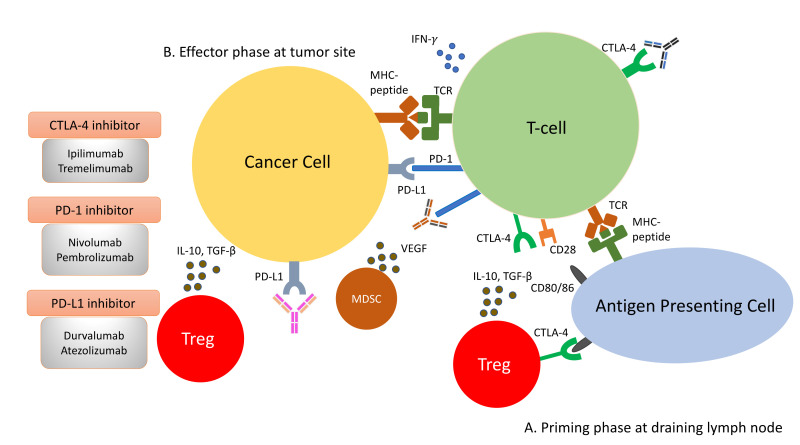
Immune response to tumour cells can be classified into two phases: the priming phase and effector phase (**A**). During priming phase, tumour antigens are detected by antigen-presenting cells, and they circulate back to lymph node to present these antigens to naïve T-cells. Naïve T-cells are activated upon encounter of its cognate antigen. A second signal is required to maintain this activation. This is mediated by the interaction of CD28 on T-cells and CD80/86 on the antigen presenting cells. When T-cells are activated, they begin to express CTLA-4 molecules, which bind to CD80/86 at higher affinity, thus removing the co-stimulation for T-cell activations and resulting in anergy (**B**). During effector phase, activated T-cells circulate back to the tumour site. When they encounter their cognate antigens, T-cells release cytotoxic granzymes and perforins to attack tumour cells. In addition to cytotoxic granules, T-cells also release IFN-*γ*, which induces PD-L1 expression on cancer cells. Binding of PD-L1 with PD-1 molecules on T-cells result in inactivation of T-cells. Apart from inactivation of T-cells from PD-1/PD-L1 interaction, the immunosuppressive milieu in the tumour microenvironment is shaped by presence of immunosuppressive cells, such as Tregs and MDSCs. They secrete immunosuppressive cytokines, such as IL-10, VEGF and TGF-β, in the tumour microenvironment. The constitutional expression of CTLA-4 at the priming site of T-cells also plays a role in dampening T-cell activation.

As opposed to CTLA-4, PD-1 is a negative regulator at the effector phase of T-cell activity at the tumour site [44]. It is an immune inhibitory receptor expressed on activated T-cells, B-cells, NK cells and dendritic cells [46]. It has two ligands: PD-L1 and PD-L2. PD-L1 is constitutively expressed on the surface on many tissues, including the tumour cells. PD-L2 is restricted to antigen-presenting cells. Upon engagement of PD-1/PD-L1, PD-1 clusters with T-cell receptors (TCR) and transiently associates with phosphatase SHP2. These microclusters decrease the phosphorylation of downstream signalling from TCR, attenuating T-cell activation [47]. Anti-PD-1/PD-L1 antibodies block the PD-1/PD-L1 interaction, enabling the reversal of T-cell anergy and restoring the anti-tumour immune response (Figure 2).

## 3. Current Evidence of Immune Checkpoint Inhibitors: Efficacy and Safety

### 3.1. ICI Monotherapy

The first evidence of ICI in advanced HCC came in 2017 in the phase I/II trial dose escalation and expansion trial, CheckMate-040, evaluating the anti-PD-1 antibody nivolumab [17]. In the dose expansion phase of the trial, 214 advanced HCC patients received nivolumab at 3 mg/kg. Notably, 20% of patients had an objective response, including three patients (1%), who achieved a complete response. Disease control rate (DCR) was 64%. The 6-month OS was 83% and the 9-month OS was 74%. Grade 3/4 treatment-related adverse events were observed in 40 (19%) patients, and grade 3/4 treatment-related serious adverse events were observed in nine (4%) patients. The most common grade 3/4 treatment-related adverse events were increased transaminase, observed in nine (4%) patients. Comparing the groups of patients with and without viral infections, the proportion of patients with symptomatic treatment-related adverse events were similar. Some patients with HCV infection had transient reductions in HCV RNA. No patients had reactivation of HBV. Taken together, this first clinical study on ICI in advanced HCC provided important insights that groomed subsequent studies of ICI in HCC. First, ICI could be used safely in patients with a background of hepatitis and cirrhosis. Secondly, the ORR and duration of response achieved by nivolumab were extraordinary in the history of systemic treatment for advanced HCC (Table 1 and Table 2). Traditionally, most sorafenib-treated HCC patients achieved stable disease but rarely achieved partial response. Complete responders were exceptional. For instance, in the SHARP trial, 71 out of 299 (24%) patients achieved a stable disease but only two patients (<1%) achieved partial response. There were no complete responders [3]. Therefore, the CheckMate-040 study ignited excitements in the use of ICI in advanced HCC. As a result of this study, the FDA granted an accelerated approval for nivolumab in sorafenib-treated advanced HCC patients in 2017. A year later, a similar phase II study, KEYNOTE-224, was published on the efficacy of another anti-PD-1 antibody, pembrolizumab, which also demonstrated clinical activity and safety in advanced HCC in the second-line setting [18].

Unfortunately, the initial hype for these two ICI was hampered by the negative results from their respective phase III trials (Table 2). In the CheckMate-459 trial, nivolumab did not demonstrate a significant improvement in OS compared to sorafenib in the first-line setting (nivolumab: 16.4 months vs. sorafenib: 14.7 months; HR: 0.85, 95%; CI: 0.72–1.02) [19]. There appeared a higher ORR favouring nivolumab compared to sorafenib (nivolumab: 17% vs. sorafenib: 7%) but it was not statistically significant. As a result, Bristol Myers Squibb voluntarily withdrew nivolumab’s indication as a post-sorafenib treatment in the U.S. market [48]. Similarly, KEYNOTE-240 failed to meet its co-primary endpoint in OS and PFS. KEYNOTE-240 was a phase III trial evaluating the efficacy of pembrolizumab vs. best supportive care in the post-sorafenib setting for advanced HCC [20]. The median PFS for pembrolizumab was 3.0 months vs. 2.8 months in the best supportive care group (HR: 0.78, 95%; CI: 0.61–0.99). The median OS was 13.9 months and 10.6 months for patients treated with pembrolizumab and best supportive care (HR: 0.78, 95%; CI: 0.61–1.00). The ORR was 18.3% in the pembrolizumab group vs. 4.4% in the best supportive care group. Although the study failed to meet its primary endpoint, the authors argued that the unanticipated better OS in the placebo group was due to the availability of effective second-line treatments (e.g., regorafenib, nivolumab) for advanced HCC, which might have diluted the effect of pembrolizumab. Indeed, the same group conducted a sensitivity analysis of the trial, demonstrating that the HR was closer to the expected value at 0.65 when subsequent anticancer treatments were considered [20].

Interestingly, in a subgroup analysis of the Asian population of the KEYNOTE-240 trial (*n* = 157), there was a trend of greater benefit with pembrolizumab compared to the overall cohort [49]. In the Asian subgroup, the median PFS was 2.8 months for the pembrolizumab group vs. 1.4 months for the placebo group (HR: 0.48; 95%; CI: 0.32–0.70). The median OS was also improved in the pembrolizumab group at 13.8 months vs. 8.3 months for the placebo group (HR: 0.55; 95%; CI: 0.37–0.80). The ORR was significantly higher in the treatment arm: 20.6% vs. 2.0%. Recently, the KEYNOTE-394 phase III randomised trial, which studied pembrolizumab in comparison to the best supportive care in the previously treated advanced HCC in the Asian population, reported a positive OS benefit [50]. The study found that treatment with pembrolizumab compared with the best supportive care resulted in a statistically significant improvement in OS (formal result pending). It also met its secondary endpoint in PFS and ORR. Together, the findings of these two trials seem to suggest that Asians may derive more benefit from anti-PD-1 treatment compared to a non-Asian population. While the full paper of KEYNOTE-394 has not been published, the Asian subgroup analysis of KEYNOTE-240 demonstrated that the major differences between Asian and non-Asian patients were in the BCLC stage (more BCLC-C in the Asian subgroup) and underlying aetiology (more viral HCC in the Asian subgroup).

### 3.2. ICI Combination: ICI and Anti-VEGF

Consistent among ICI regimens is the extraordinary duration of response for those who have an objective response. Active research has been conducted to search for biomarkers that can identify excellent responders, albeit without success to date. Previous trials have also failed to demonstrate that PD-L1 expression is a reliable marker for anti-PD-1/L1 ICI [17,18]. As a result, effort has shifted to explore ICI combination strategies to increase the OS, ORR and duration of response in the entire patient group. One such strategy is to combine ICI with an anti-VEGF. There is good scientific rationale behind this combination strategy: Firstly, the anti-VEGF agent normalises the torturous blood vessels secondary to neovascularisation within the tumour, enabling better infiltration of ICI at the tumour site. Secondly, anti-VEGF counteracts the VEGF secreted by Tregs in the tumour microenvironment, restoring effective immune surveillance [52].

IMbrave150 was the first phase III randomised trial testing the ICI-based combination regimen (atezolizumab, an PD-L1 inhibitor combined with bevacizumab, an anti-VEGF) that yielded a positive finding over a sorafenib-controlled arm [23,24]. In IMbrave150, patients with advanced HCC were randomised in a 2:1 ratio to receive atezolizumab–bevacizumab (*n* = 336) or sorafenib (*n* = 165). The study was stopped early at the first interim analysis due to the demonstration of significant improvements in median PFS (HR: 0.65, 95%; CI: 0.53–0.81; *p* < 0.001) and median OS (HR: 0.66, 95%; CI: 0.52–0.85; *p* < 0.001). Notably, the study demonstrated an unprecedented ORR in the advanced HCC of 30%, including 8% of complete responder, and a median OS of 19.2 months. There were no alarming safety events observed with the atezolizumab–bevacizumab combination. Serious adverse events occurred more frequently with atezolizumab–bevacizumab (38%) than with sorafenib (30.8%), but no specific events were responsible for the increased incidence of serious adverse events in the atezolizumab–bevacizumab group. The most common grade 3/4 treatment-related adverse event was hypertension (15.2%), consistent with the previous known safety profile of bevacizumab. Grade 3/4, increased in aminotransferase, occurred in 7% of the treatment group vs. 5% in the sorafenib group. As a result of IMbrave150, atezolizumab–bevacizumab became the new standard of treatment for advanced HCC, as recommended by international HCC guidelines [53,54]. Other similar trials were underway, evaluating the combination of ICI and multi-kinase inhibitor, including the LEEP-002 trial on the pembrolizumab–lenvatinib combination (NCT03713593) and the COSMIC-312 trial on the atezolizumab–cabozantinib combination (NCT03755791).

### 3.3. ICI Combination: ICI and ICI

Another ICI combination strategy is to target two immune checkpoints simultaneously. This can be achieved by blocking both PD-1/PD-L1 and CTLA-4. This strategy releases the “brake” from T-cell activation in both the priming phase and effector phase. The addition of CTLA-4 inhibitor restores the costimulatory signal triggered by CD80/86-CD28 binding on T-cells and eliminates Tregs via antibody-dependent cytotoxicity to enhance the T-cells activation [44].

CheckMate-040 cohort 4 was a phase I/II study that evaluated the efficacy and safety of ipilimumab (anti-CTLA-4) and nivolumab (anti-PD-1). This combination is the only dual ICI combination that has been approved by the FDA so far for sorafenib-treated advanced HCC [21]. There were three randomised arms in the trial. Arm A: nivolumab 1 mg/kg plus ipilimumab 3 mg/kg, administered every three weeks (four doses), followed by nivolumab 240 mg every two weeks; arm B: nivolumab 3 mg/kg plus ipilimumab 1 mg/kg, administered every three weeks (four doses), followed by nivolumab 240 mg every two weeks; arm C: nivolumab 3 mg/kg every two weeks plus ipilimumab 1 mg/kg every six weeks. Amongst the three arms, arm A attained the highest ORRs (A: 32%, B: 27% and C: 29%), complete response rates (A: 8%, B: 6% and C: 0%) and median OS (A: 22.8, B: 12.5 and C: 12.7 months). While the combination therapy offered higher ORR compared to the nivolumab monotherapy, this strategy was associated with higher toxicity and with higher rates of adverse events. Grade 3/4 adverse events were 55%, 29% and 31% in arms A, B and C, respectively. Although arm A (with the highest ipilimumab dose) had the highest adverse event rates, the type of adverse events was comparable across treatment arms for patients. About 50% of patients received glucocorticoid therapy for immune-related adverse events. However, rechallenging with either nivolumab or ipilimumab after experiencing immune-related adverse events appeared to be safe, and none of the patients experienced a recurrence of the event. Intriguingly, in a subsequent exposure-response analysis of this study, the same group demonstrated that there was a positive association between OS and ipilimumab exposure but not nivolumab exposure [55]. Even though cross-trial comparisons were intrinsically difficult, the OS and ORR in arm A represented the highest observed amongst all regimes approved for advanced HCC in this setting.

Tremelimumab (anti-CTLA-4) and durvalumab (anti-PD-L1) were another combination being tested in the management of advanced HCC. In a randomised expansion phase I/II study reported recently, 332 patients who progressed on, were intolerant to, or refused sorafenib were assigned to four arms. Arm A: tremelimumab 300 mg plus durvalumab 1500 mg for one dose followed by durvalumab 1500 mg every four weeks; arm B: durvalumab monotherapy 1500 mg once every four weeks; arm C: tremelimumab monotherapy 750 mg once every four weeks for seven doses, then once every 12 weeks; arm D: tremelimumab 75 mg once every four weeks plus durvalumab 1500 mg once every four weeks for four doses, followed by durvalumab 1500 mg once every four weeks [22]. Amongst the four arms, arm A attained the highest ORR at 24%, with one patient achieving a complete response. Similarly, median OS was the highest with arm A at 18.7 months. The median OS for arms B, C and D were 15.1, 13.6 and 11.3 months, respectively. Median PFS were 2.2, 2.1, 2.7 and 1.9 months, respectively, for arms A, B, C and D. The safety analysis demonstrated that arm C (tremelimumab monotherapy) had the highest rate of grade 3/4 treatment-related adverse events, at 43.5%. Arm A, which had the highest median OS and ORR, comparatively had less grade 3/4 treatment-related adverse events, at a rate of 35.1%. The most common grade 3/4 treatment-related adverse event was increased transaminase. Treatment-related adverse events requiring a systemic steroid were higher for tremelimumab-containing arms (arm A: 24.3%, arm B: 9.9%, arm C: 26.1% and arm D: 24.4%). Overall, the safety profile appeared favourable and manageable, with only 6% to 13% of patients across arms discontinuing the treatment due to treatment-related adverse events. In the translational part of the study, it showed that the proliferative CD8+Ki67+ T-cell count was associated with radiological response, and the count was highest amongst patients who received a higher dose of tremelimumab. This correlation supported an underlying mechanism of enhanced immune activation with a CTLA-4 inhibitor.

In the corresponding phase III trial HIMALAYA, which was presented in ASCO GI 2022, the combination of tremelimumab and durvalumab demonstrated a superior OS benefit, compared to sorafenib in the first-line setting for advanced HCC [51]. In the trial, 393 patients were randomised in the combination arm (tremelimumab 300 mg for one dose and durvalumab 1500 mg every four weeks) and 389 patients were randomised in the sorafenib arm. The median OS was superior in the combination arm, at 16.4 months vs. 13.8 months in the sorafenib arm (HR: 0.78, 95%; CI: 0.65–0.92). The median PFS was not significantly different (3.8 months vs. 4.1 months; HR: 0.90, 95%; CI 0.77–1.05), which is consistent with previous findings that the onset of immunotherapy tends to be slower than multi-kinase inhibitors. ORR was 20.1% in the combination arm (3.1% complete response and 17.0% partial response) vs. 5.1% in the sorafenib arm (0% complete response and 5.1% partial response). The safety profile of tremelimumab and durvalumab was favourable and consistent with earlier phase studies. Grade 3/4 treatment-related adverse events were 25.8% in the combination arm and 36.9% in the sorafenib arm, respectively. About 20% of patients who developed immune-mediated adverse events required high-dose steroids. The most common immune-related adverse events were hepatic related (e.g., increased aminotransferase), at about 4%. Altogether, only about 6% of patients discontinued treatment due to an immune-related toxicity.

Taken together, the addition of anti-CTLA-4 with anti-PD-1 immunotherapy produces a statistically significant improvement in OS with a manageable safety profile, providing an alternative option for advanced HCC patients who are not suitable for multi-kinase inhibitors or anti-VEGF, such as in the context of increased bleeding risks commonly found in advanced HCC patients.

## 4. The Paradox of Immune Checkpoint Inhibitors and NASH-HCC

### 4.1. Are NASH-HCC Poor Responders to ICI?

While the extraordinary ORR and OS offered by ICI have been heralded as the game changer in the management of advanced HCC, a recent paper published in *Nature* titled ‘NASH Limits Antitumour Surveillance in Immunotherapy’, has provided shocking evidence of the potential detrimental effect of ICI in NASH-HCC [31]. The study first demonstrated that the frequency of CD8+PD1+ T-cells was specifically increased in NASH-HCC in mice models. Because of these high numbers of T-cells in NASH-HCC, it was initially thought that the anti-PD-1 treatment may serve as an effective treatment. Surprisingly, when NASH-HCC mice were treated with anti-PD-1 therapy, none of the tumour regressed. Instead, there was an increased incidence of liver fibrosis and liver cancer. However, in non-NASH-HCC mice, a shrinkage of tumours was observed after anti-PD-1 therapy. These findings suggested that anti-PD-1 therapy failed to reinvigorate CT8+PD1+ T-cells to perform effective immunosurveillance. Instead, anti-PD-1 therapy promoted tissue damage and malignant changes. To further verify the function of CD8+PD1+ T-cells, the group depleted NASH-mice of CD8+PD1+ T-cells and found a significant drop in the incidence of HCC in these mice. Treating anti-PD-1 therapy prophylactically in NASH-mice increased CD8+PD1+ T-cells, aggravated liver damage and heightened the incidence of HCC. This evidence provided strong support of the carcinogenic role of CD8+PD1+ T-cells in NASH-mice.

The group took their study further to examine if similar findings could be extrapolated to human-NASH. Using single-cell RNA sequencing, they demonstrated that the CD8+PD1+ T-cells in patients with NASH shared similar gene expression patterns with those in NASH-mice. Subsequently, the group conducted a meta-analysis of three published phase III trials (CheckMate-459, KEYNOTE-240 and IMbrave150) comprising a total of 1656 patients to study the effect of immunotherapy in HCC according to the underlying aetiologies. The meta-analysis revealed that immunotherapy did not improve OS in non-viral HCC (HR: 0.92, 95%; CI: 0.77–1.11), whereas those with viral HCC derived survival benefits from immunotherapy (HR: 0.64, 95%; CI: 0.48–0.94). To isolate the effect of anti-PD-L1 immunotherapy with respect to the underlying aetiology of liver damage, the group investigated two additional small cohorts of NAFLD-HCC patients. Consistent with their hypothesis, NAFLD-HCC patients had a shortened OS after immunotherapy, as compared to patients with other aetiologies. In the first validation cohort (*n* = 130), the median OS was 5.4 months for NAFLD-HCC vs. 11.0 months for HCC of non-NAFLD aetiology; in the second validation cohort (*n* = 118), the median OS was 8.8 months vs. 17.7 months. Collectively, this study signalled a proposition that immunotherapy might not confer beneficial effects in NAFLD/NASH-HCC.

### 4.2. Progression from NASH to NASH-HCC

In the past few years, new knowledge has been added to our understanding on the molecular mechanisms that drive the transition from NAFLD/NASH to NASH-HCC. These findings support a parallel and multiple-hits paradigm of immune cell-hepatocyte interactions that contribute to the development NASH-HCC [56,57,58]. Firstly, CD8+ T-cells and NKT-cells were demonstrated to cooperatively induce liver damage and carcinogenesis via interaction with hepatocytes in a NASH-mouse model [58]. In addition, the metabolic activation of intrahepatic NKT-cells caused steatosis via the secretion of LIGHT. These interactions resulted in the downregulation of metabolic machinery in hepatocytes, thereby increasing oxidative stress and the activation of procarcinogenic pathways, such as LTβR and canonical NF-κB signalling, promoting a NASH-to-HCC transition [58]. Secondly, platelet aggregations as the initial inflammatory process, in the context of steatosis, were demonstrated to drive NASH to the NASH-HCC transition [59]. This phenomenon was mediated via the interactions between the platelet-specific glycoprotein Ib-α (GPIbα) with Kupffer cells. Antiplatelet therapy was demonstrated to reduce intrahepatic platelet accumulation and the frequency of platelet–immune cell interaction, attenuating NASH and NASH-related HCC in the mouse model [59]. Indeed, a recently published meta-analysis (*n* = 2,389,019) demonstrated that the use of aspirin was associated with significantly lower HCC risk (RR: 0.61, 95%; CI: 0.51–0.73) [60]. Thirdly, it was noted that in NASH-mice or patients with NASH, there was a distinctive group of residential CD8+ T-cells in the liver that demonstrated an auto-aggressive killing of cells in an MHC-class-I independent fashion [61]. Mechanistically, IL-15 induced FOXO-1 downregulation and CXCR6 upregulation, which together rendered liver-resident CXCR6+CD8+PD1+ T-cells susceptible to metabolic stimuli, such as the acetate released by hepatocytes with steatosis. These CXCR6+CD8+ T-cells then secreted tumour necrosis factor (TNF) and injured hepatocytes. In addition, these activated CXCR6+CD8+PD1+ T-cells caused hepatocyte cell death through a Fas ligand-dependent apoptosis. This auto-aggressive mechanism of cell killing mediated via CXCR6+CD8+PD1+ T-cells was fundamentally distinct from that by antigen presentation in protective adaptive immunity. It has been postulated that this auto-aggression of CXCR6+CD8+PD1+ T-cells in the liver is responsible for the chronic liver damage in NASH and may be implicated in the development of HCC (Figure 1).

### 4.3. Unresolved Questions in NASH-HCC and ICI

The meta-analysis conducted by Pfister et al. raised some important key questions [31]. Contrary to the preclinical findings of enhanced tumour progression in NASH-mice treated with ICI, the ORRs of non-viral HCC patients receiving nivolumab or pembrolizumab appeared similar to patients with viral HCC in the range of 20% [62] (Table 3). Subgroup analyses for patients treated with atezolizumab and bevacizumab demonstrated similar improvement in PFS, regardless of the underlying causes of HCC [23,24]. The favourable ORRs and PFS in the non-viral HCC patients without improvement in OS have led to several alternative hypotheses. Firstly, there was a fundamental limitation of the meta-analysis, in which it included a heterogeneous population of non-viral HCC patients. Non-viral HCC includes a wide spectrum of aetiologies, such as NASH, alcoholic steatohepatitis, cryptogenic cirrhosis, primary biliary cirrhosis, and autoimmune hepatitis, etc. A lack of stratification of the underlying liver disease would make it difficult to tease out the effect of ICI on NASH-HCC. Secondly, concluding ICI was ineffective in non-viral HCC, based solely on the HR without looking at the comparator arm, is inappropriate. The control arm of the two included trials (IMbrave150 and CheckMate-459) in the meta-analysis was sorafenib. The HRs demonstrated that, relative to sorafenib, ICI demonstrated OS benefits in viral HCC but not in non-viral HCC (viral HCC—HR: 0.64 vs. non-viral HCC—HR: 0.92) [63]. It is commonly thought that non-viral HCC patients in general respond better with molecular-targeted agents (e.g., sorafenib) because they often present with favourable clinical–pathological characteristics, such as the absence of cirrhosis [64,65,66]. In particular, non-viral HCC patients treated with sorafenib demonstrated a much longer median OS compared to viral HCC in the IMbrave150 trial [23] (Table 2). Therefore, the more appropriate postulation would be that the longer survival benefits of sorafenib in non-viral HCC diluted the benefits of ICI, resulting in a seeming lack of benefits of ICI in non-viral HCC. Alternatively, it could be that the survival benefits of ICI are much more pronounced in viral HCC, as observed in the Asian populations, where viral HCC is predominant [49]. Thirdly, it is uncertain whether there were differences in the access to downstream treatments in patients with non-viral HCC in the control group, which could have driven better outcomes. Fourthly, the sequence of treatments might have impacts on the outcome in patients with non-viral HCC, as observed in the management of other malignancies. In a small retrospective study evaluating 25 patients with BRAF-mutant melanoma, the treatment with immunotherapy after a BRAF inhibitor resulted in higher ORR, compared to giving immunotherapy before a BRAF inhibitor (43.8% vs. 0%). The OS, however, was not different [67]. To answer these questions, well-designed clinical trials will be needed to draw definite conclusions on the effectiveness of ICI in NASH-HCC.

## 5. In Search of Biomarkers Predictive of Immune Checkpoint Inhibitor Response

To date, no robust biomarkers predicting responses to ICI in patients with HCC have been found. Unlike the non-small cell carcinoma of lung (NSCLC) [68,69], where immunohistochemical staining of PD-L1 expression predicts response to anti-PD-L1 therapy, its role in HCC remains uncertain. In the dose expansion cohort of CheckMate-040, 174 patients had evaluable PD-L1 expression; 9 (26%) out of 34 patients with PD-L1 expression ≥ 1% had an objective response to nivolumab, compared to 26 (19%) out of 140 patients with a PD-L1 expression < 1% [17]. On the contrary, the KEYNOTE-224 study, which examined second-line pembrolizumab over best supportive care, demonstrated that a positive PD-L1 expression (combined positive score) was associated with a higher ORR and PFS [18]. There were also interests in looking into whether microsatellite instability (MSI) or tumour mutation burden (TMB) correlated with the response to ICI, but these were hampered by the low prevalence of MSI and TMB-high HCCs [70,71].

Antidrug antibodies (ADA) have been studied as biomarkers to predict responses to ICI. Administration of any biologic agents to humans is likely to be recognised by the immune system and trigger a humoral response, resulting in ADA formation [72]. The presence of ADA can reduce the bioavailability of the drugs and may result in decreased anti-tumour activity. The reported range of ADA varied amongst ICI, from 54% for atezolizumab, to as low as 1.5% for pembrolizumab [72]. Thus far, the clinical relevance of ADA is not entirely clear; however, exploratory analyses appeared to support the hypothesis of a reduced efficacy of atezolizumab in ADA-positive patients. For example, in IMbrave150, exploratory analyses demonstrated that the subset of patients who were ADA-positive by week six derived less OS benefit from the atezolizumab–bevacizumab combination, compared to those who were ADA-negative (HR: 0.93 vs. HR: 0.39) [73]. Similarly, in the OAK trial which evaluated second-line atezolizumab against docetaxel in metastatic NSCLC, exploratory analyses demonstrated that patients who were ADA-positive by week four appeared to have reduced OS benefit, as compared to patients who tested negative [73,74].

Renewed interest in the gene expression assay has grown to predict ICI response. Recently, Haber et al. performed a transcriptomic analysis on 111 tumour samples from patients with HCC treated with the single-agent anti-PD1 [30]. They noted that the response to frontline anti-PD1 was associated with significant upregulation in IFN-γ signalling and antigen presentation machinery. Using differential gene expression analysis, they constructed an 11-gene signature, which was predictive of response, as well as PFS and OS in advanced HCC. The same 11-gene signature was validated in three independent cohorts of melanoma, lung cancer and head and neck squamous cell carcinoma patients. In these three cohorts, patients with high expression were associated with significant increase in response and PFS. In another study evaluating tumour gene expression profile from CheckMate-040 using RNA-sequencing technique, a 4-gene inflammatory signature (PD-L1, CD8A, LAG3 and STAT1) was associated with improved ORR and OS [29]. While these results were encouraging, they would need to be validated in a prospective setting.

## 6. Future Directions

Immunotherapy has opened a new era of management in advanced HCC. In comparison with other approved treatment options, immunotherapy has offered an unprecedented ORR up to 35% [21]. The IMbrave150 study has set a new standard for the first-line treatment of advanced HCC with atezolizumab and bevacizumab, resulting in a record-high OS at 20 months [23,24]. The recently published results from the HIMALAYA trial demonstrated similar OS benefits with the combination strategy using two ICIs (tremelimumab and durvalumab), providing an alternative treatment option for patients with contraindications to anti-VEGF agents [51]. HCC is no longer considered a dismal disease with only a few months of survival once diagnosed. Despite all these encouraging results with immunotherapy, the recently published paper by Pfister et al. drew attention to a potential linkage between NASH and a reduced response to ICI in patients with HCC. This linkage signalled a word of caution in the use of immunotherapy, without considering the underlying aetiology of HCC, and underscored the necessity of stratification in future trials according to the cause of liver disease.

It is still too early for a change in clinical practice in HCC based on the underlying aetiology of HCC. The undeniable improvement in OS derived from the combination of atezolizumab–bevacizumab in the IMbrave150 study and tremlimumab–durvalumab in the HIMALAYA study, coupled with the limitations of the meta-analysis described above, provided support in maintaining ICI combination strategy as the first-line standard treatment of HCC. However, it is important to keep in mind the underlying aetiology of HCC and that immunotherapy may not be effective for NASH-HCC. As soon as progressive disease is observed, a quick switch to second-line treatment is warranted.

Looking into the future, NASH is fast becoming the most common cause of cirrhosis in most regions of the world and is a rapidly increasing cause of HCC in the West [2]. Appropriately designed trials specifically addressing NASH-HCC are desperately needed. While the beneficial role of immunotherapy in the management of HCC is undeniable, reliable biomarkers to predict responses to ICI are needed to select patients who would most likely (or unlikely) benefit from treatment. With more treatment options and combination strategies available for the treatment of advanced HCC, future trials will also need to address what would be the best treatment sequence in advanced HCC, with regards to underlying liver diseases and other clinical–pathological factors.

Furthermore, there has been some evidence to suggest that epidemiological factors, such as ethnicity, lifestyle, and diet may influence the response to ICI [75]. Of note, increasing data suggest that the gut microbiome not only plays a key role in hepatocarcinogenesis, but may also play a role in modulating the anti-tumour response following immunotherapy [76]. In a group of patients (*n* = 249) with advanced NSCLC, renal cell carcinoma and urothelial carcinoma who received ICI, patients who received antibiotics had a significantly shortened PFS and OS than those who did not receive antibiotics [77]. Metagenomics analysis in the same study demonstrated that a low level of the bacterium *Akkermansia muciniphila* correlated with poor response to ICI. Oral supplementation of *A. muciniphila* in mice who received faecal microbiota transplantation from non-responding patients restored the efficacy of ICI [77]. A phase II trial is currently recruiting patients with refractory HCC to receive a combination of nivolumab, tadalafil and oral antibiotics vancomycin, to see whether this combination can produce a synergistic anti-tumour effect by altering the gut microbiome.

## 7. Conclusions

The introduction of ICI has changed the treatment landscape of advanced HCC. Combination strategies with an anti-VEGF or an ICI offer unprecedented ORR and survival benefits. Recent studies have begun to shed light on our understanding of the impact of the underlying aetiology of chronic liver disease, such as NASH, on the response to ICI. In addition, biomarkers and gene signatures are being explored to predict responders to ICI. Further studies incorporating these factors are needed to address the optimal treatment strategies for the different subgroups of HCC patients.

## Figures and Tables

**Table 1:** Summary of main outcomes among systemic therapies (multi-kinase inhibitors) approved for advanced HCC.

Study	Year	Phase	N	Line of Tx	Tx	CR (%)	ORR (%)	mPFS (Months)	mOS (Months)	HR ^X^ (95%CI)
SHARP [3]	2008	III	602	First	Sorafenib	0	2	5.5	10.7	0.69 (0.55–0.87)
Asia-Pacific [4]	2009	III	271	First	Sorafenib	0	3	2.8	6.5	0.68 (0.50–0.93)
REFLECT [9]	2018	III	476	First	Sorafenib	<1	9	3.7	12.3	-
REFLECT [9]	2018	III	478	First	Lenvatinib	1	24	7.4	13.6	0.92 (0.79–1.06)
RESORCE [10]	2017	III	573	Second	Regorafenib	1	11	3.1	10.6	0.63 (0.50–0.79)
CELESTIAL [11]	2018	III	707	Second	Cabozantinib	0	4	5.2	10.2	0.76 (0.63–0.92)
REACH-2 [12]	2019	III	542	Second	Ramucirumab	0	5	2.8	8.5	0.71 (0.53–0.95)

CI: Confidence interval; CR: Complete response; HR: Hazard ratio; mOS: Median overall survival; mPFS: Median progression-free survival; N: Number of patients; ORR: Objective response rate; Tx: Treatment; ^X^ HR for mOS.

**Table 2:** Summary of main outcomes among systemic therapies (immunotherapy) approved for advanced HCC.

Study	Year Published	Phase	Number of Patients	Line of Tx	Tx	Control	CR (%)	ORR (%)	mPFS (Months)	mOS (Months)	HR ^x^ (95% CI)
IMbrave 150 [23,24]	2018	III	501	First	Atezolizumab + Bevacizumab	Sorafenib	8 vs. 1	30 vs. 11	All: 6.8 vs. 4.3 HBV: 6.7 vs. 2.8 HCV: 8.8 vs. 5.8 Non-viral 7.1 vs. 5.6	All: 19.2 vs. 13.4 HBV: 19.0 vs. 12.4 HCV: 24.6 vs. 12.6 Non-viral: 17.0 vs. 18.0	All: 0.66 (0.52–0.85) HBV: 0.58 (0.40–0.83) HCV: 0.43 (0.25–0.73) Non-viral: (0.68–1.63)
CheckMate 459 [19]	2021	III	743	First	Nivolumab	Sorafenib	4 vs. 1	16 vs. 7	3.8 vs. 3.9	16.4 vs. 14.7 *	0.85 (0.72–1.02)
KEYNOTE-240 [20]	2019	III	413	Second	Pembrolizumab	BSC	2 vs. 0	18 vs. 4	3.0 vs. 2.8	13.6 vs. 10.6 *	0.78 (0.61–1.00)
KEYNOTE-240 (Asian cohort) [49]	2019	III	157	Second	Pembrolizumab	BSC	-	21 vs. 2	2.8 vs. 1.4	13.8 vs. 8.3	0.55 (0.37–0.80)
CheckMate 040 (cohort 4) ^+^ [21]	2020	I/II	50	Second	Nivolumab + Ipilimumab	-	8	32	-	22.2	-
CheckMate 040 (dose expansion cohort) [17]	2017	I/II	214	Mixed	Nivolumab	-	1	20	4.0	NR	-
KEYNOTE-224 [18]	2018	II	104	Second	Pembrolizumab	-	1	17	4.9	12.9	-
Study 22 ^++^ [22]	2021	I/II	75	Second	Tremelimumab + Durvalumab	-	1	24	2.2	18.7	-
HIMALAYA [51]	2022	III	393	First	Tremelimumab + Durvalumab	Sorafenib	3 vs. 0	20 vs. 5	3.8 vs. 4.1	16.4 vs. 13.8	0.78 (0.65–0.92)

BSC: Best supportive care; CI: Confidence interval; HR: Hazard ratio; mOS: Median overall survival; mPFS: Median progression-free survival; NR: Not reached; ORR: Objective response rate; Tx: Treatment; * did not reach statistical significance; ^+^ results from the Nivolumab 1 mg/kg plus Ipilimumab 3 mg/kg Q4wk arm; ^++^ results from the T300+D arm; ^x^ HR for mOS.

**Table 3:** Summary of objective response rates according to underlying liver diseases in major trials.

Study	Aetiology (Proportion)	Tx	Control	ORR (%) (Tx Arm)	ORR (%) (Control Arm)
IMbrave150 [23]	HBV (48%)	Atezolizumab + Bevacizumab	Sorafenib	32	8
IMbrave150 [23]	HCV (21%)	Atezolizumab + Bevacizumab	Sorafenib	30	21
IMbrave150 [23]	Non-viral (31%)	Atezolizumab + Bevacizumab	Sorafenib	27	9
CheckMate 459 [19]	HBV (31%)	Nivolumab	Sorafenib	19	8
CheckMate 459 [19]	HCV (23%)	Nivolumab	Sorafenib	17	7
CheckMate 459 [19]	Non-viral (45%)	Nivolumab	Sorafenib	12	7
KEYNOTE-224 [18]	HBV (21%)	Pembrolizumab	-	24	-
KEYNOTE-224 [18]	HCV (25%)	Pembrolizumab	-	8	-
KEYNOTE-224 [18]	Non-viral (55%)	Pembrolizumab	-	30	-
CheckMate 040 (dose expansion) [17]	HBV (24%)	Nivolumab	-	14	-
CheckMate 040 (dose expansion) [17]	HCV (23%)	Nivolumab	-	20	-
CheckMate 040 (dose expansion) [17]	Non-viral * (53%)	Nivolumab	-	22	-

ORR: Objective response rate; Tx: Treatment; * uninfected untreated+uninfected progressor.
